# A qualitative exploration of contraceptive use and discontinuation among women with an unmet need for modern contraception in Kenya

**DOI:** 10.1186/s12978-021-01094-y

**Published:** 2021-02-09

**Authors:** Susan Ontiri, Lilian Mutea, Violet Naanyu, Mark Kabue, Regien Biesma, Jelle Stekelenburg

**Affiliations:** 1 Jhpiego Corporation, Johns Hopkins University Affiliate, Nairobi, Kenya; 2 Department of Health Sciences, Global Health, University Medical Centre Groningen/University of Groningen, Groningen, The Netherlands; 3 USAID Kenya and East Africa, Nairobi, Kenya; 4 School of Arts and Social Sciences, Moi University, Eldoret, Kenya; 5 Jhpiego Corporation, Johns Hopkins University Affiliate, Baltimore, MD USA; 6 Department of Obstetrics and Gynecology, Leeuwarden Medical Centre, Leeuwarden, The Netherlands

**Keywords:** Contraceptive discontinuation, Contraceptive side effects, Counseling, Quality of care, Kenya, Unmet need for family planning

## Abstract

**Background:**

Addressing the unmet need for modern contraception underpins the goal of all family planning and contraception programs. Contraceptive discontinuation among those in need of a method hinders the attainment of the fertility desires of women, which may result in unintended pregnancies. This paper presents experiences of contraceptive use, reasons for discontinuation, and future intentions to use modern contraceptives.

**Methods:**

Qualitative data were collected in two rural counties in Kenya in 2019 from women with unmet need for contraception who were former modern contraceptive users. Additional data was collected from male partners of some of the women interviewed. In-depth interviews and focus group discussions explored previous experience with contraceptive use, reasons for discontinuation, and future intentionality to use. Following data collection, digitally recorded data were transcribed verbatim, translated, and coded using thematic analysis through an inductive approach.

**Results:**

Use of modern contraception to prevent pregnancy and plan for family size was a strong motivator for uptake of contraceptives. The contraceptive methods used were mainly sourced from public health facilities though adolescents got them from the private sector. Reasons for discontinued use included side effects, method failure, peer influence, gender-based violence due to covert use of contraceptives, and failure within the health system. Five reasons were provided for those not willing to use in the future: fear of side effects, cost of contraceptive services, family conflicts over the use of modern contraceptives, reduced need, and a shift to traditional methods.

**Conclusion:**

This study expands the literature by examining reasons for contraceptive discontinuation and future intentionality to use among women in need of contraception. The results underscore the need for family planning interventions that incorporate quality of care in service provision to address contraceptive discontinuation. Engaging men and other social influencers in family planning programs and services will help garner support for contraception, rather than focusing exclusively on women. The results of this study can inform implementation of family planning programs in Kenya and beyond to ensure they address the concerns of former modern contraception users.

## Background

Use of contraceptive methods allows spacing of pregnancies or limiting family size, enabling individuals and couples to fulfill their fertility desire by choosing if and when to become pregnant. Contraceptive use not only has positive effects on health-related outcomes, such as improved maternal and child health [1] but also improves schooling and economic outcomes for girls and women [2]. Global trends have shown an increase in contraceptive uptake, however, many women, approximately one out of three, discontinue their method within a year [3, 4]. Contraceptive discontinuation is an important determinant of contraceptive prevalence, as well as unintended pregnancies, and other demographic impacts as it increases the unmet need for family planning (FP). Several studies have found that contraceptive abandonment and failure contribute substantially to the total fertility rate, unwanted pregnancies, and induced abortions [3–5]. Analysis of data from 36 developing countries revealed that over one-third of unintended pregnancies resulted from women who had discontinued the use of contraception [5]. Unintended pregnancies have negative consequences on the health and well-being of women and their families as they can lead to maternal morbidities and even death. Besides, it is documented that children born from unintended pregnancies are: less likely to be breastfed, more likely to be stunted, at risk of a lack of parental love, and at higher risk of child mortality than children from wanted pregnancies [6].

An analysis of Demographic and Health Surveys conducted by Curtis et al. demonstrated that women’s socio-demographic characteristics—age, education, place of residence, and economic status—are the determinants associated with contraceptive discontinuation [7]. Even though studies indicate that women with higher levels of education and those residing in urban residences are more likely to discontinue their initial method, additional analyses reveal that these women are more likely to switch than stop after discontinuing a method [7–9]. This could be because they are enlightened on their contraceptive choices and will discontinue and switch if a particular method does not suit them since they can also easily access the contraceptive services due to shorter distances to health facilities.

Researchers continue to investigate why a woman or a couple would discontinue the use of modern contraception while still in need. Past studies show side effects and health concerns have been the main causes of contraceptive discontinuation [3, 4, 10]. Indeed, side effects account for more than half of the reasons for discontinuing contraceptives while still in need [9, 11].

Kenya has implemented a strong national family planning (FP) program since it was launched in 1967 [12]. Over the past five decades, the country has developed FP/reproductive health policies, strategies, and guidelines and implemented programs aimed at increasing access and utilization of modern contraceptive methods among women of reproductive age and supporting men's involvement. These efforts have borne fruit; the current data estimates a contraceptive prevalence rate of 62.8%, which is mostly driven by the use of modern methods at 60.7% [13]. However, more than one-third of all pregnancies in Kenya are unintended and one in three women discontinue use of contraceptives by 12 months [14]. Like other countries, the main reason cited in Kenya for discontinuation is side effects, predominantly side effects associated with hormonal contraception [14]. Studies have linked poor quality of care, particularly inadequate counseling on side effects with contraceptive discontinuation [4, 15]. For instance, data from round 5 to round 7 of Kenya’s Performance Monitoring and Accountability 2020 surveys indicate a glaring gap in the quality of FP services provided in health facilities. Only two-thirds of women were informed about side effects by service providers, with slightly more than half being informed about what to do in case of side effects [13, 16, 17].

Whereas the predictors of contraceptive counseling have been established by several quantitative studies [3, 4, 18], there is a paucity of information to understand the lived-in experiences of women who discontinue the use of contraceptives while still in need. This paper reports qualitative results from in-depth interviews and focus group discussions with discontinuers. The interviews and discussions explored experiences with previous use of modern contraceptives, reasons for discontinuation, and future intention to use contraceptives among discontinuers.

## Methods

### Study design and setting

A cross-sectional qualitative study was conducted as part of a formative assessment in a 24-month longitudinal study on evaluating the dynamics of contraceptive use, discontinuation, and switching in Kenya. The longitudinal study is being conducted in Kitui and Migori, rural counties in Kenya. The two counties have a diverse method mix; Migori’s mCPR is mostly driven by long-acting reversible contraceptives, at 72% while in Kitui, short-term methods are more popular, at 64% [14]. Details of the longitudinal study, including the study setting, have been published elsewhere [19]. Ten public health facilities, five in each county were purposively selected based on high FP caseload. The 10 facilities were located in 10 different sub-counties. Routine service statistics revealed that these facilities provided the highest number of contraceptive services in their respective sub-counties. Out of the ten facilities, 2 were county hospitals, 5 sub-county hospitals, 2 health centers, and 1 dispensary. The consolidated criteria for reporting qualitative research (COREQ) was used in this paper [20]. The completed checklist is available in Additional file 1.

### Study participants

Since the main objective of this study was to explore the experience with contraceptive use and discontinuation among discontinuers, participants who met the following inclusion criteria were selected: women of reproductive age between 15 and 49 years of age, who were sexually active, did not desire pregnancy, and had been but were currently not using modern contraception. The men who were interviewed to explore their perspective on contraceptive discontinuation were purposively selected since they were spouses of the women who met the inclusion criteria. Data collection included FGDs with adolescent mothers aged 15–19 years and women over 20 years and IDIs with couples and adolescent girls. Recruitment of study participants stopped once data saturation was achieved, that is when no new information was derived from the interviews and focus group discussions. In total, 42 data collection sessions (12 FGDs and 30 IDIs) were conducted with 135 study participants-105 in FGDs and 30 in IDIs. (Table 1).

**Table 1:** Number of data collection sessions conducted

Type of qualitative participant	Method of data collection	No. of sessions	Average number of participants per session	No. of participants
Women (20–49 years)	FGD	6	9	56
Adolescent mothers (15–19 years)	FGD	6	8	49
Adolescent girls (15–19 years)	IDIs	10	1	10
Married couples (18–49 years), interviewed separately	IDIs	20	1	20
Total		42		135

### Recruitment strategy

The study team selected community health volunteers (CHVs) who were providing health information including family planning to households within the catchment area of the study facilities. The CHVs were trained on the inclusion criteria and thereafter, mobilized and screened community members within their catchment area before referring them to the study staff who contacted, further screened, and recruited those eligible into the study. For couples, the CHV would approach the woman first to establish eligibility, before contacting the spouse. Both partners had to agree to participate before inclusion in the study.

### Data collection

Data collection was conducted from May to July 2019. The data collection team was comprised of 10 research assistants, (seven females and three males) who had undergraduate training in Anthropology or Sociology. The team was selected based on their experience conducting qualitative studies. They further received an additional 5-day refresher training before data collection. They worked under the supervision of the lead author. Respondents were not known to the interviewers before the data collection sessions. Written consent was obtained from the participants to conduct and audio-record the data collection sessions. The time and place of the interviews were determined based on the convenience of the participants. The venue for the FGD data collection sessions was community halls while the IDIs were conducted at the participants’ homes. All participants were aware that the study was being conducted to explore their perspective and experience with contraceptive use and discontinuation as part of a formative assessment to improve the quality of family planning services provided.

Semi-structured topic guides covering FP topics for the various audiences were developed and piloted before use. The FGD guide included open-ended prompts related to knowledge and perception of contraceptives, use of FP with their community, and reasons for contraceptive discontinuation, including influencers. The study had IDI guides for the adolescent girls (15–19 years) and for married couples (18–49 years), husbands and wives were interviewed separately. The former group was asked about their knowledge and perceptions around sexual and reproductive health and contraceptive use, experience using contraceptives, and contraceptive discontinuation. The married couples shared their knowledge, perception, and decision-making experiences using contraceptives; FP use and discontinuation; and couple involvement in contraceptive use and discontinuation. The file showing the topic guides used in this study is provided in Additional file 2.

Two trained interviewers were present at each FGD—one as a session moderator and the other as a note-taker. For the IDIs, only one trained moderator was present for the conversation. No observer was present during data collection. The FGDs and interviews were conducted in local dialect (Kamba and Dholuo) and Swahili. All the interviews were audio-recorded, and field notes were taken for each focus group session. The interview sessions lasted between 30 and 90 min. The data collection team debriefed after the end of each session. Interim findings were discussed weekly by the team and interview guides were modified and revised as needed. At the end of data collection, no new themes were emerging and data saturation had been achieved.

### Data analysis

The digital recordings of IDIs and FGDs were transcribed verbatim, translated into English, and analyzed using NVivo 11. Data were analyzed thematically following the approach of Braun and Clarke to identify, analyze, and report patterns within the data [21]. Coding and theme development were directed by the content of the data (inductively) [21]. A final agreed thematic framework was applied to all interviews. Transcripts were not returned to participants in advance of coding. Data analyses were performed by two researchers (VN and SO) with in-depth knowledge of qualitative analysis who were supported by two analysts to ensure timely coding and validation of the coding frame. The team identified themes from reading and rereading the transcripts, noting any similarities and differences between and within participants’ accounts. The preliminary findings were shared with some of the study participants for validation.

### Ethical considerations

This study was guided by a protocol that was approved by the Kenya Medical Research Institute Institutional Review Board and the Johns Hopkins Bloomberg School of Public Health Institutional Review Board. Participants gave informed written consent/assent to participate in the study. Protection and confidentiality of participants was ensured through conducting data collection sessions in private settings, maintaining confidentiality, and limiting access to study information to only authorized personnel.

## Results

The demographic characteristics of the 135 study participants are shown in Table 2. The majority of the participants were adolescents and youth aged 15–24 years at 51%, had primary education 53%, were farmers 32%, and had one to two children (Table 2). The findings from the two study sites were comparable, with no major differences.

**Table 2:** Demographic characteristics of the participants

Variable	Categories	n	%^a^
Age (years)	15–19	59	44
	20–24	9	7
	25–34	35	26
	35–49	32	24
Education level	None	5	4
	Primary	72	53
	Secondary	55	41
	College	2	1
	University	1	1
Occupation	Employed	1	1
	Farming	43	32
	Business	20	15
	Not working	71	53
Number of children^b^	0	10	8
	1–2	71	57
	3–4	28	22
	5 +	16	13

^a^Proportions may not add to 100 because of rounding

^b^For couples, only wives’ information is presented

Study findings are provided in four themes below: (1) motivation for modern contraceptive use; (2) sources and decision-making for previous contraceptive used; (3) barriers to sustained use of contraceptives; and (4) future intention to use contraceptives.

### Motivation for modern contraceptive use

The study explored the participant’s motivation for use of a contraceptive prior to discontinuation. Generally, there was strong consensus among all the study participants that the reasons for using contraceptives were to plan for the number of children they wished to have, and prevent pregnancy. Adolescent participants further noted that the greatest motivation for using contraceptives was to prevent pregnancy so as to pursue studies; they wanted to avoid unplanned pregnancies that might result in having to drop out of school and take on parental responsibilities they had not envisioned.

Economic reasons appeared to be the major impetus for use of contraceptives by adolescent mothers, older women, and married couples, as most participants shared similar sentiments on the need to have children they can manage to raise as illustrated by the following quote:

*“We are able to space out the children and able to provide the right foods to the children so that they can be healthy because our incomes are low.”* (FGD, Female).

Many participants reported that their motivation for use of contraceptives was to space their pregnancies to allow the healthy growth of children so they could get enough attention, nutrition, and care from their parents. A few married women noted, where couples were experiencing marital conflict, women used contraceptives to avoid getting additional children that they would need to support on their own.

### Sources and decision-making for previous contraceptive used

The majority of participants interviewed indicated that they got their contraceptive method from public health facilities. Some, especially adolescents, got their contraceptive methods from private facilities, specifically chemists or pharmacists. Most older respondents indicated that they had opted for injectables and implants, while use of pills was mainly mentioned by adolescents.

*“I bought my pills from the pharmacy shop in town”* (IDI, Adolescent, Female).

The study findings revealed that before using contraception, most women sought the opinions of partners, peers, or family friends. For adolescent mothers, their mothers were mentioned as helpful in decision-making and accessing contraceptives. Most partners were involved in decision-making about uptake of FP before initiation of a method, while some were engaged after the FP method was started. However, some female participants stated that they had used contraception covertly due to non-supportive spouses or relatives, particularly the in-laws who threatened to report them to their partners.

### Barriers to sustained use of contraception

The study further explored the reasons why women did not continue using a contraceptive method yet they still had a need for contraception. Reasons for discontinued use of contraceptives were manifold; five main sub-themes emerged: side effects, method efficacy, peer influence, gender-based violence, and health system factors.

#### Side effects of contraceptives

Across all the study groups, side effects resulting from use of contraception were repeatedly mentioned among the reasons for discontinuation. The leading side effect was irregular bleeding patterns presenting as menorrhagia (heavy menstrual bleeding) or amenorrhea (absence of menstrual bleeding). This was mainly experienced from the use of hormonal methods, and in particular injectables and implants. For example:

*“When I used the three-months injection, I was bleeding excessively. Sometimes I would feel dizzy while walking. The bleeding would even continue for a month without stopping. So, I decided to stop using it.”* (IDI, Female).

Heavy bleeding was cited to interfere with the participants’ social and economic lifestyle. The majority of the female participants who reported increased bleeding indicated that they were unable to carry out their economic activities since they were weak as a result of the increased menstrual flow. Another recurrent consequence of the increased bleeding was the interference with their sexual life:

*“The reason I chose to stop using depo is for one reason. Sometimes my husband may have the desire to get intimate with you but you cannot, because of the bleeding. Whenever I want us to get intimate he declines because it is so much blood that is why he told me to try quitting it.”* (IDI, Female).

On the contrary, some respondents reported that the absence of menstrual bleeding was what triggered discontinuation since they did not know whether they were still fertile or were pregnant.

*“When I started using implants, my periods did not come for eight months, then it came back only for two days and disappeared again. I decided to stop using a contraceptive since I was always wondering whether I was pregnant.”* (FGD, Adolescent).

Other side effects that led to discontinuation, albeit less frequently mentioned across the various study groups, included weight changes, dizziness, and low sexual libido.

“*My friend who was using the one for three years told me she stopped because she didn’t have an appetite for having sex, so it was raising issues between her and her husband.”* (FGD, Adolescent).

Some study participants observed that experiences from other women influenced contraceptive use or discontinuation. Several FGD participants indicated that women discontinued the use of contraceptive methods after learning about side effects experienced by their friends. This prompted even those who were not experiencing the same to discontinue out of fear.

#### Contraceptive method efficacy

Contraceptive efficacy was a concern mentioned mostly by married couples. Respondents reported method failure whereby women got pregnant unexpectedly while still on a contraceptive method:

*“One year after using an implant, I started becoming sick. When I went back to the hospital, I was tested and the results came out that I was four months pregnant, and at the same time I still had the implant in my arm.”* (FGD, Female).

*“I have a friend; she was using the one for 3 months. After sometime, she was shocked that she was pregnant. So, she decided that she will not use it because even if you use it you still get pregnant.”* (FGD, Adolescent).

Several participants revealed that they decided to discontinue use of contraceptives after learning about cases of method failure among women who were using similar methods. On several instances, inconsistent use of contraceptive, especially short-term methods, that resulted in pregnancies were reported as method failure by some participants:

*“The one for three months confused her a lot, it came to end without her knowing and she forgot to go back to the clinic for another injection. She became pregnant and then it surprised her. We had tried using it for a long time and I told her that she was using a method of a shorter duration and when it ended she became pregnant without planning.”* (IDI, Male).

#### Covert use of contraception resulting in gender-based violence

Covert use of contraception was common due to lack of spousal support for use of a modern method. Across all the study groups, the participants shared their experiences or cases of other women who discontinued contraceptive use because their partners learned that they were using it covertly. Cases of gender-based violence directed at women by their partner after learning their use of modern contraceptive methods, further solidified their resolve to discontinue as illustrated by this experience:

*“Another woman in our village went and got an implant without her husband’s knowledge. When the husband learned of this, he took a knife and removed it from her arm. This made my friends and me afraid, so we decided to just remove it for fear of what our husbands would do if they find out.”* (FGD, Female).

#### Health system factors as a barrier to continuation

Health care system factors were repeatedly mentioned as reasons for discontinuation. Stock-outs of preferred methods during contraceptive initiation or resupply prompted women to either take alternative methods or leave without one. Provider bias that resulted in women taking up methods that they did not approve of came up as a sub-theme particularly by younger women, as shown in the quote below:

*“I told him [the provider] I wanted depo and he said that the government does not advise the use of injection, and he refused to put it on me. He convinced me to take up an implant, which I did, but I went to another facility to have it removed.”* (FGD, Female).

There were mixed experiences regarding FP counseling, particularly on side effects. Several respondents noted that they got adequate counseling by the health care providers during the initiation of a method; however, some mentioned that they were not informed of any potential side effects that could result from use of contraception.

*“When I started using them, the doctor explained to me about the advantages and disadvantages of the various methods of family planning, such that, I know the goodness and effects of the method I am using.”* (FGD, Female).

### Future intentionality to use contraception

The study explored whether the respondents would consider using modern contraceptives again. Several respondents indicated willingness to use at some time, but some were hesitant. Those who would consider using an FP method again said they would consult widely, select a method with fewer side effects, and one with a longer duration. For those who were doubtful and not considering using FP, five reasons were provided.

First, there were fears about negative side effects. Women indicated that the fear of experiencing another side effect after discontinuation led them to decide not to take up any other modern method despite the counseling that they got from health care workers who were advising them on method switching. One woman shared her experience:

*“These medicines bring problems. I stayed with the one injection for a while and every time I would feel sickly, weak, back pains at all times, bleeding from Monday to Monday. I came to the hospital and asked them to remove it. They asked me what the problem was, that they will give me another one, but I did not want one. So that is why I stopped using.”* (FGD, Female).

Second, cost was cited as a barrier for continued use. Respondents indicated that the direct and indirect costs associated with uptake of contraceptive services hindered their intention to use. The cost barrier was mainly mentioned for short-term methods that require frequent resupply at facilities, hence, women had to make multiple visits to the facility. Several concerns were also raised regarding the removal of intrauterine contraceptive devices or implants after experiencing side effects. An important issue that participants highlighted was the cost incurred for the removal of a method, which caused women to fear the selection of another method in case they experienced side effects with that method.

*“If you go to the facility before the expiry date, you are asked to pay 200 shillings, regardless of the side effects experienced. I wonder why they charge for removal yet they gave it for free. After that one fears to take up another method.”* (FGD, Female).

Lastly, FP use caused conflicts in families. Women indicated lack of support from their partners and relatives impeded their intention to use contraception. It was evident that even though the women felt a need to space or limit their family size, that decision was mainly made by their partners. Other women, who had previously used the method covertly and had been discovered by their spouses or relatives, mentioned they could not use the method for fear of gender-based violence. This quote buttresses the point:

*“My husband threatened to beat me also if he ever found me using a method. This was after he had observed a disagreement between our neighbors (couple), over the discreet use of contraceptives that ended up with the lady being hit by her husband. I decided to stop using to avoid such an occurrence.*” (IDI, Female).

## Discussion

This qualitative study aimed to explore the dynamics of contraceptive use and discontinuation among women with unmet need for contraceptives in the rural counties of Migori and Kitui, Kenya. A large and diverse group of adolescents, women, and couples who reported contraceptive discontinuation while still in need of a method provided insights on their experiences, perspectives with contraceptive use and reasons for discontinuation. Direct quotes of study participants about their experiences with FP use that culminated in discontinuation have been presented to deepen understanding of participants’ experiences [22]. From the study findings, it is evident that all the respondents chose to use contraceptives with the conviction that by using a modern method, they would be able to prevent pregnancy or plan when to have children, determine how far apart they want their children to be, and when to stop having children. However, this desire was not fully realized as they discontinued use of the contraceptives while still in need, which added to the pool of women of reproductive age with unmet need for FP.

There were numerous challenges faced by women using contraceptives that prompted them to discontinue their use. As noted in prior studies, side effects play a major role in reported decisions to discontinue [4, 23, 24]. Our study revealed that the most common side effect leading to contraceptive discontinuation were changes in users’ bleeding patterns, findings which are consistent with studies conducted across different parts of the world [18, 25, 26]. Irregularity of bleeding negatively impacts the well-being of women, mainly due to the social consequences, which could explain the low tolerance with contraception when such side effects are encountered. Studies have revealed that women, especially in the sub-Saharan region, believe that menstrual bleeding is a sign of fertility, hence any change that leads to reduced or no bleeding is frowned upon [27, 28]. Conversely, increased bleeding impacts women’s socio-economic activities and sexual relationship with their partners [28, 29].

Our findings thus provide strong support for addressing side effects experienced by women through management when they occur or being provided options for method switching to ensure the women continue to harness the full benefits of contraception. This can be achieved by conducting client follow-up by service providers to periodically assess the level of satisfaction with the contraceptive method while addressing issues that might prompt clients to discontinue. Proper counseling of clients, and their partners, is crucial to promote continuation with use of modern contraceptive methods as the users are made aware of the contraceptive’s mechanism of action, possible side effects, and what to do when they experience side effects. Helping women understand typical bleeding changes associated with their contraceptive methods could lead to greater acceptance of the changes, increased method uptake, improved satisfaction, and higher continuation rates [30]. Therefore, capacity building of health care providers on contraceptives should not just focus on the technical skills on insertion and removal (particularly for long-term methods), but also on contraceptives’ mechanisms, how they work, to ensure that providers are well versed on the potential side effects for each method. This is supported by evidence from studies in Madagascar and Ghana that revealed providers were not well informed on the physiological effects of contraception and how to manage side effects [4]. This resulted in inadequate counseling of women experiencing the side effects; women were counseled to switch to another method instead of being reassured that side effects would settle down over time or being offered medication to control some side effects [4]. This could be attributed to inadequate training content on side effects. A recent review of FP counseling, training, and reference materials revealed that bleeding changes are insufficiently addressed in capacity building resources and counseling tools for health care providers [29]. This is alarming, considering that the leading reason for discontinuation has been changes in bleeding pattern. Skilled counseling for side effects, particularly bleeding irregularities, can only be achieved if training materials for health care providers incorporate this information, information that will improve the quality of counseling by health care providers.

Contraceptive method failure was one of the reasons for discontinuation in this study. Method failure is a factor of either failure of a method to work as expected or incorrect/inconsistent use of a method by the user. In low- and middle-income countries, 74 million unintended pregnancies occur annually, of which a sizable share, 30%, are due to contraceptive failure among women using some type of contraceptive method [31]. Each contraceptive method has a Pearl Index number that reflects pregnancy rates during perfect and typical use, with use of long-term method conferring higher efficacy than short-term methods [32]. Whereas all contraceptive methods have some degree of failure, even during perfect use, failure rates can be reduced when individuals are sensitized on the proper use of contraception to ensure the method is used correctly and consistently. Provision of clear information about the risks and benefits of all available methods is crucial in facilitating informed contraceptive choice so women can make an educated choice for their preferred methods, which may reduce discontinuation.

Other reasons for contraceptive discontinuation, such as lack of support from partners and other social networks, are also corroborated in researches previously conducted in Kenya [28, 33]. In our study, the decision to use or not use contraceptives was still primarily made by men. Although women made solo decisions on FP, they were heavily influenced by their spouses’ preference and would stop using if they thought it would bring marital conflicts. Opposition to contraceptive use by husbands appears to stem from the fear of side effects and the perception that women who use FP are more likely to be promiscuous. Additionally, Kenya being a highly patriarchal society, decision-making around the desired number of children mainly lies with the male partner. FP programs have mainly targeted women with information to promote uptake since they are the ones who face the risk of pregnancy and childbirth. Unfortunately, these programs have left out men, who are in most instances, the decision-makers in male-dominated societies, like most countries in the sub-Saharan region [34]. The findings from this study reveal the power dynamics when it comes to a couple’s decision to use contraception. This underscores the need to meaningfully involve men in FP programs by informing them of the health, economic, and social benefits realized from proper and consistent use of contraception so they can optimize use of FP services. Demand generation strategies that employ the use of positive deviants, satisfied users, and other key influencers, such as mothers-in-law, may lead to an increase in contraceptive uptake and enhance continuation.

This study indicates that the costs associated with consistent use of FP methods hinder their continued use. Promoting uptake of LARC methods will address the cost associated with the use of short-term method—LARCs have been shown to be more cost-effective and do not require frequent visits to facilities [35].

Our study also revealed punitive measures women faced, especially those on LARCs, when they wanted to switch to another method before its expiration. Allowing for method switching is indicative of strong FP programs that have an adequate range of methods and a flexible environment to meet women’s needs. Due to the health and social concerns that contraceptive use may confer on individuals, women may try different methods before settling for their preferred option. The health system should have a supportive policy environment that accommodates such needs of women by: instituting guidelines that prohibit penalization for method switching; addressing commodity stock-outs and ensuring sufficient method mix through increased financing of FP programs; and sensitizing providers on the importance of method switching by women who are not satisfied with their methods. Additional studies are needed to document the implications of frequent method switching on commodity security in countries that continue to face widespread stock-outs of contraceptive methods.

The study’s main strength was documenting the experiences of contraceptive use and discontinuation among discontinuers themselves. However, qualitative studies have limitations related to validity, subjectivity, and reliability. To address these issues, efforts were made to increase the rigor and trustworthiness of the findings through the selection of participants with a range of backgrounds and experiences with the guidance and supervision of experts, as well as external review. Information was not collected on the number of eligible participants who refused to participate in the study. Despite this, our study benefits from including a large number of participants, diverse in terms of age, gender, ethnicity, and location, and utilizing different data collection methodologies (FGDs and IDIs) to enrich the findings.

## Conclusions

Our study, conducted in two rural counties in Kenya, revealed a number of important findings regarding factors influencing contraceptive use and discontinuation. The participants in this study had a common motivation for using contraception, to avoid pregnancies, however, side effects were a major hindrance in continued use of contraception. Covert use of contraception resulted in discontinuation when it was discovered and, in some instances, led to gender-based violence. Decision-making on contraception, method to use, and the number of children to have, was jointly done by couples or made by the husband. Reasons for discontinuation, specifically on side effects, were influenced by the husbands.

As contraceptive use in a population increases, success in avoiding unintended pregnancies depends less on initial contraceptive uptake and more on effective and persistent use. Enhanced efforts are needed to design and implement programs that focus on contraceptive discontinuation among women with unmet need for FP. Health care providers offering FP services should be well versed with the mechanism of action for the various contraceptive methods, and incorporate quality of care in the provision of contraceptive services. Additionally, contraception technological advancement is urgently needed to expand the method mix and to develop methods that have fewer side effects and side effects that can be more easily tolerated. This will go a long way in promoting continuation of contraceptive use, as indicated by a majority of our study participants who were willing to consider future use of contraception methods with fewer side effects. Findings from this study, as well as other studies, confirm the importance of engaging men and other social influencers in FP programs by educating them on the socio-economic and health benefits of family planning and dispelling any myths and misconceptions to create a social environment that supports use of modern contraception.

## Supplementary Information

**Additional file 1:** Consolidated criteria for reporting qualitative research completed checklist.

**Additional file 2:** Qualitative interview guides.

## Data Availability

The data used and analysed during the current study are available from the corresponding author on reasonable request.

## Abbreviations

COREQ: Consolidated criteria for reporting qualitative studies
CHV: Community health volunteers
FGD: Focus group discussions
FP: Family planning
IDI: In-depth interviews
LARC: Long-acting and reversible contraceptive
TFR: Total fertility rate
