# Supplementary material for: A qualitative exploration of contraceptive use and discontinuation among women with an unmet need for modern contraception in Kenya
Source: Reprod Health. 2021 Feb 9;18:33. doi: 10.1186/s12978-021-01094-y (PMC7871615; doi:10.1186/s12978-021-01094-y)
Supplement: Supplementary file 2 — Additional file 2: Qualitative interview guides. [file 12978_2021_1094_MOESM2_ESM.docx]

# Tool 1 - FGD guide for adolescent mothers (15-19 years)

**Knowledge and perceptions around SRH and contraceptive use**

1. Where do boys and girls in your community learn about pregnancy and how to prevent it?
2. What are some of the cultural beliefs and practices in this community with focus on pregnancy among adolescents?
3. What is your understanding of contraceptives?
4. Who in your opinion can use contraceptives? Please explain
5. What are some of the local beliefs that affect whether people of your age use contraceptives or not in this community?

**Contraceptive discontinuation**

1. In your opinion, do we have instances where young people discontinue using contraceptive methods*?*
2. What are some of the reasons why young people switch from one method to another’s?
3. What are some of the factors that prevent you and other girls in this community from using contraceptives after delivery of the first child? Please explain
4. In your opinion, what prevents adolescent girls from using contraceptives?
5. What needs to be done to help young women and girls continue to use contraceptives as required?
6. Any additional comments?

# Tool 2 - IDI guide for adolescents (15-19 years)

**Knowledge and perceptions around SRH and contraceptive use**

1. What is your understanding of sexual and reproductive health?
2. In terms of pregnancy prevention, what are some of the things people of your age do to protect themselves from getting pregnant?

**Experience using contraceptives**

1. What is your understanding of contraception?
2. Would you please share with me if you have ever used contraceptives?
3. How old were you when you started using contraceptives?
4. What made you consider using contraceptives? Who influenced your decision?

**Contraceptive discontinuation**

1. When did you last use that method? What made you stop using the method?
2. Are you considering using any method to delay or avoid getting pregnant in the near future?

# Tool 3 - FGD guide for women of reproductive age (20 – 49 years)

**General information of reasons for contraceptive use**

1. Briefly share with us on the contraceptives used by women and girls in this community?
2. What are some of the reasons why women use contraceptives?

**Factors that influence discontinuation**

1. Are there instances in this community where women stop using contraceptives while in need of one?
2. Could you briefly share with us some of the reasons that make women stop using the method?
3. When women discontinue a method, are there instances where they discontinue a method with the aim of switching to another? Please explain

**Decision making processes in case of discontinuation**

1. Generally, what steps do women take before discontinuing a method?
2. What are some of steps that need to be taken to prevent women from discontinuing contraceptive use despite them being in need of the same?
3. In your opinion what services should be provided at the facility at the time of initiation that will help women who opt to discontinue a method to be motivated to take up another method?

# Tool 4: IDI guide for married heterosexual couples

**General knowledge, perception and experiences around using contraceptive methods**

1. What does the use of contraceptive mean to you?
2. In your community, are there any beliefs and perceptions that influence the use or no use of family planning?
3. What are some of the reasons why women and girls stop using contraceptives when they don’t want to get pregnant?

**FP use and discontinuation**

1. Have you or your partner ever used any modern contraceptive?
2. What reasons made you consider using contraceptives?
3. For how long did you use contraception(s)? When did you stop using a contraceptive method?
4. What were the reasons that informed the discontinuation?
5. Who influenced the decision to discontinue?

**Couple involvement in decision making around contraceptive use and discontinuation**

1. Which methods are couples in this community most likely to use?
2. In this community how are decisions around contraceptive use mostly arrived at in relationships? What about in your marriage?
3. Would you say that using contraception is mainly a woman’s decision, their partner's decision, or both of them decide together? Please explain
4. (For wives): To what extent did you involve/inform your partner on your decision to use contraceptives?
5. What is your experience with discontinuation? What was the role of your partner in stopping the use of the method? Which method did your partner prefer? What were the reasons behind this? Did you agree to their reasons? How did you go about this issue?
6. Would you ever consider using a contraception method again? (Probe: if no, ask why, if yes, when? Which method?)
